# Off-axis rotor in *Enterococcus hirae* V-ATPase visualized by Zernike phase plate single-particle cryo-electron microscopy

**DOI:** 10.1038/s41598-018-33977-9

**Published:** 2018-10-23

**Authors:** Jun Tsunoda, Chihong Song, Fabiana Lica Imai, Junichi Takagi, Hiroshi Ueno, Takeshi Murata, Ryota Iino, Kazuyoshi Murata

**Affiliations:** 1 The Graduate University for Advanced Studies (SOKENDAI), Kanagawa, 240-0193 Japan; 2 National Institute for Physiological Sciences, Okazaki, Aichi 444-8585 Japan; 3 Department of Chemistry, Graduate School of Science, Chiba University, Inage, Chiba, 263-8522 Japan; 4 Institute for Protein Research, Osaka University, 3-2 Suita, Osaka, 565-0871 Japan; 5 Department of Applied Chemistry, University of Tokyo, Tokyo, 113-8656 Japan; 6 JST, PRESTO, Inage, Chiba, 263-8522 Japan; 7 Institute for Molecular Science, Okazaki, Aichi 444-8787 Japan

## Abstract

*Eh*V-ATPase is an ATP-driven Na^+^ pump in the eubacteria *Enterococcus hirae* (*Eh*). Here, we present the first entire structure of detergent-solubilized *Eh*V-ATPase by single-particle cryo-electron microscopy (cryo-EM) using Zernike phase plate. The cryo-EM map dominantly showed one of three catalytic conformations in this rotary enzyme. To further stabilize the originally heterogeneous structure caused by the ATP hydrolysis states of the V_1_-ATPases, a peptide epitope tag system was adopted, in which the inserted peptide epitope sequence interfered with rotation of the central rotor by binding the Fab. As a result, the map unexpectedly showed another catalytic conformation of *Eh*V-ATPase. Interestingly, these two conformations identified with and without Fab conversely coincided with those of the minor state 2 and the major state 1 of *Thermus thermophilus* V/A-ATPase, respectively. The most prominent feature in *Eh*V-ATPase was the off-axis rotor, where the cytoplasmic V_1_ domain was connected to the transmembrane V_o_ domain through the off-axis central rotor. Furthermore, compared to the structure of ATP synthases, the larger size of the interface between the transmembrane a-subunit and c-ring of *Eh*V-ATPase would be more advantageous for active ion pumping.

## Introduction

V-ATPase is a rotary enzyme that forms a large membrane protein complex of approximately 800 kDa. It globally plays a role in regulating cellular functions and the subcellular environments by ion transportation using energy obtained from ATP hydrolysis. This enzyme is known to perform physiological functions, such as proteolysis^1^, bone resorption^2^, and membrane potential formation^3^. Dysfunctions of V-ATPase cause several critical diseases, such as tumors^4^, osteoporosis^5^, and distal renal tubular acidosis^6^. V-ATPase consists of two major catalytic components: the V_1_ and V_o_ domains. The cytoplasmic V_1_ domain including ATPases transfers ATP-hydrolyzed energy to the transmembrane V_o_ domain by rotating its central rotor. The architecture of V-ATPase is similar to those of A- and F-ATPases, despite they conversely catalyze ATP synthesis using the energy of the ion gradient across the membrane under physiological conditions^7–10^.

The overall structure of V-ATPase has been reported in *Thermus thermophilus* (*Tt*) and *Saccharomyces cerevisiae* (*Sc*)^9–11^. Although *Tt* V-ATPase can pump H^+^ by ATP hydrolysis and therefore termed V/A-ATPase, it usually functions as an ATP synthase^12^. The architecture of V/A-ATPase consists of nine components including two peripheral stalks, though it contains a relatively smaller c-ring consisting of 24 transmembrane helices compared to other V-ATPases. Cryo-electron microscopy (cryo-EM) maps had revealed two of three rotational catalytic conformations^9^. Recently, the minor third catalytic conformation was also identified in the higher resolution maps^10^. In contrast, *Sc*V-ATPase possesses one more peripheral stalk and several auxiliary subunits (H, and C-subunit) that are not observed in *Tt*V/A-ATPase^11^. *Sc*V-ATPase also functions as a H^+^ pump using ATP hydrolysis energy. The high-resolution cryo-EM maps of these ATPases revealed different structural conformations depending on three rotational catalytic states with biased stoichiometry^10,11^. The transmembrane regions commonly indicate that the conserved glutamates in individual c-subunits continuously interact with the conserved arginine in the long and tilted double α-helices of the a-subunit during rotation of the c-subunit ring. This suggests a H^+^ transport mechanism where the transfer is performed between these two residues^13^. The overall structures of these V-ATPases together with their three catalytic conformations provide the snapshots of the dynamics for H^+^ transportation coupled with ATP hydrolysis.

By contrast, the entire structure of *Eh*V-ATPase structure has remained unknown. *Eh*V-ATPase is an ATP-driven ion pump in *Enterococcus hirae*, particularly involved in the transport of Na^+^ ^14^. Except for the large size of the c-ring consisting of 40 transmembrane helices, the subunit composition of *Eh*V-ATPase is the same as that of *Tt*V/A-ATPase. The fact that ion selectivity is determined by only differences of several amino acids in the c-ring^15^ supports the structural similarity between these ATPases. The atomic structures of individual subunits except for the d- and a-subunits in the V_o_ domain and peripheral stalks have been reported by X-ray crystallography for *Eh*V-ATPase^15,16^. The three catalytic conformations in the V_1_ domain have also been identified using a non-hydrolysable ATP analog^17,18^. However, the molecular coupling between the V_1_ and V_o_ domains and pumping mechanism of ions in the V_o_ domain remains unclear.

In this study, we report the first complete structure of *Eh*V-ATPase determined by Zernike phase plate (ZPP) cryo-EM and single-particle analysis, which suggests a mechanism of ion transportation via the transmembrane V_o_ domain coupled with ATP hydrolysis in the V_1_ domain. Furthermore, we applied peptide epitope (PA) tag system^19,20^ to stabilize the originally heterogeneous structure caused by the ATP hydrolysis states of the V_1_ part by stopping the rotation of the central rotor with Fab bound to a braking site. The PA tag sequence introduced to the d-subunit which is a part of the central rotor in *Eh*V-ATPase and the bound Fab unexpectedly revealed the different conformation and consequently identified one minor catalytic conformation. Interestingly, these catalytic conformations with and without Fab conversely coincided with those of the minor state 2 and the major state 1 in *Tt*V/A-ATPase, respectively. The most prominent feature was the off-axis rotor in *Eh*V-ATPase, where the cytoplasmic V_1_ motor and transmembrane V_o_ pump were connected to the off-axis rotor subunits.

## Results

### Sample purification

**Figure 1:**
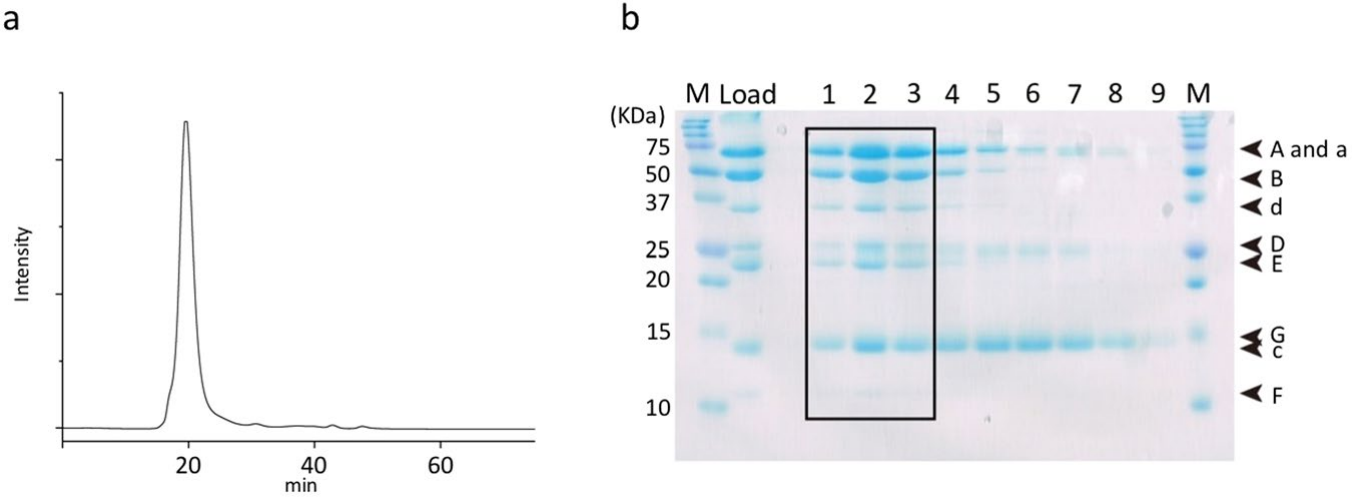
Sample purification. (**a**) Size exclusion chromatography of r-*Eh*V-ATPase. (**b**) 16% SDS-PAGE of r-*Eh*V-ATPase. Nine components consisted of all subunits were identified. Molecular markers are shown on both sides (M). Fraction numbers are labeled as 1–9 on the top. The samples of boxed fractions (1–3) were used for subsequent cryo-EM analysis. The full-length gel is presented.

Recombinant *Eh*V-ATPase (r-*Eh*V-ATPase) purified with a nickel-nitrilotriacetic acid column was applied for size-exclusion column chromatography (Fig. 1a). The fractions of the peak were subjected to SDS-PAGE, revealing bands that included all 9 components of r-*Eh*V-ATPase (Fig. 1b). This result showed the intact multicomplex of r-*Eh*V-ATPase was purified. Additionally, the recombinant complex was functionally intact, where DCCD, an inhibitor of c-ring rotation in the transmembrane V_o_ domain, blocked ATP hydrolysis in the cytoplasmic V_1_ domain^21^.

### Cryo-electron microscopy

**Figure 2:**
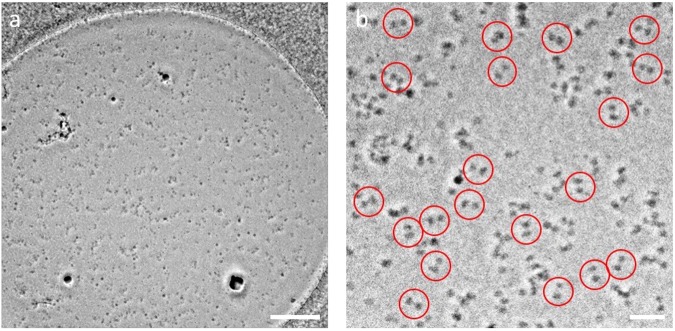
Image of r-*Eh*V-ATPase by ZPP cryo-EM. (**a**) ZPP cryo-EM image of ice-embedded r-*Eh*V-ATPase particles. Scale bar: 200 nm. (**b**) Higher magnification image of r-*Eh*V-ATPase particles. Representative particles are labeled with red circles. Scale bar: 50 nm.

**Figure 3:**
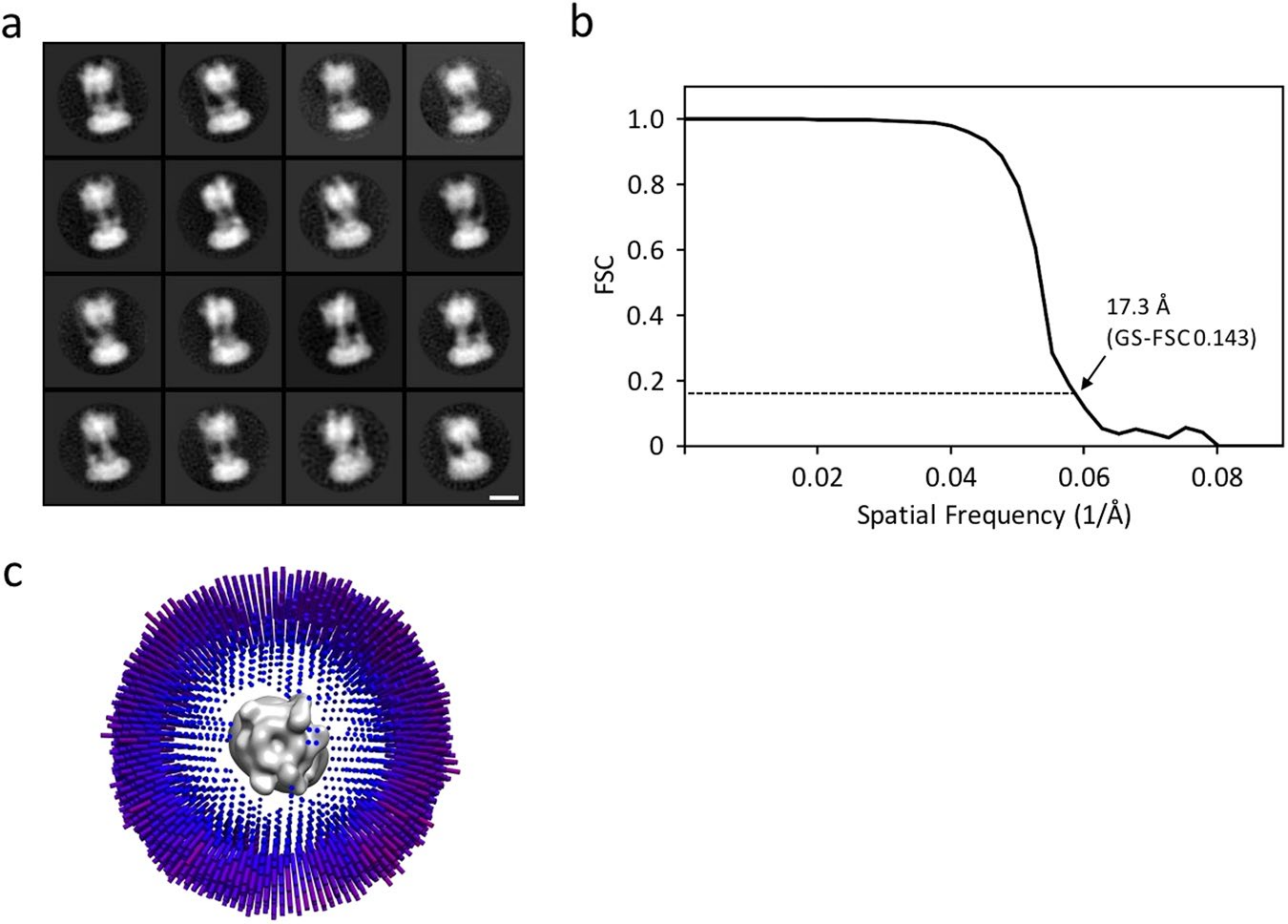
Single-particle analysis of r-*Eh*V-ATPase by ZPP cryo-EM. (**a**) Representative 2D class averages of r-*Eh*V-ATPase particles. 2D class averages were calculated from ~120,000 particles. Scale bar: 10 nm. (**b**) Gold-standard Fourier shell correlation (GS-FSC) curve for the final 3D reconstruction of r-*Eh*V-ATPase. Resolution was estimated as 17.3 Å using GS-FSC criteria. (**c**) Angular distribution of individual images in the final 3D reconstruction, showing unbiased image sampling was achieved.

**Figure 4:**
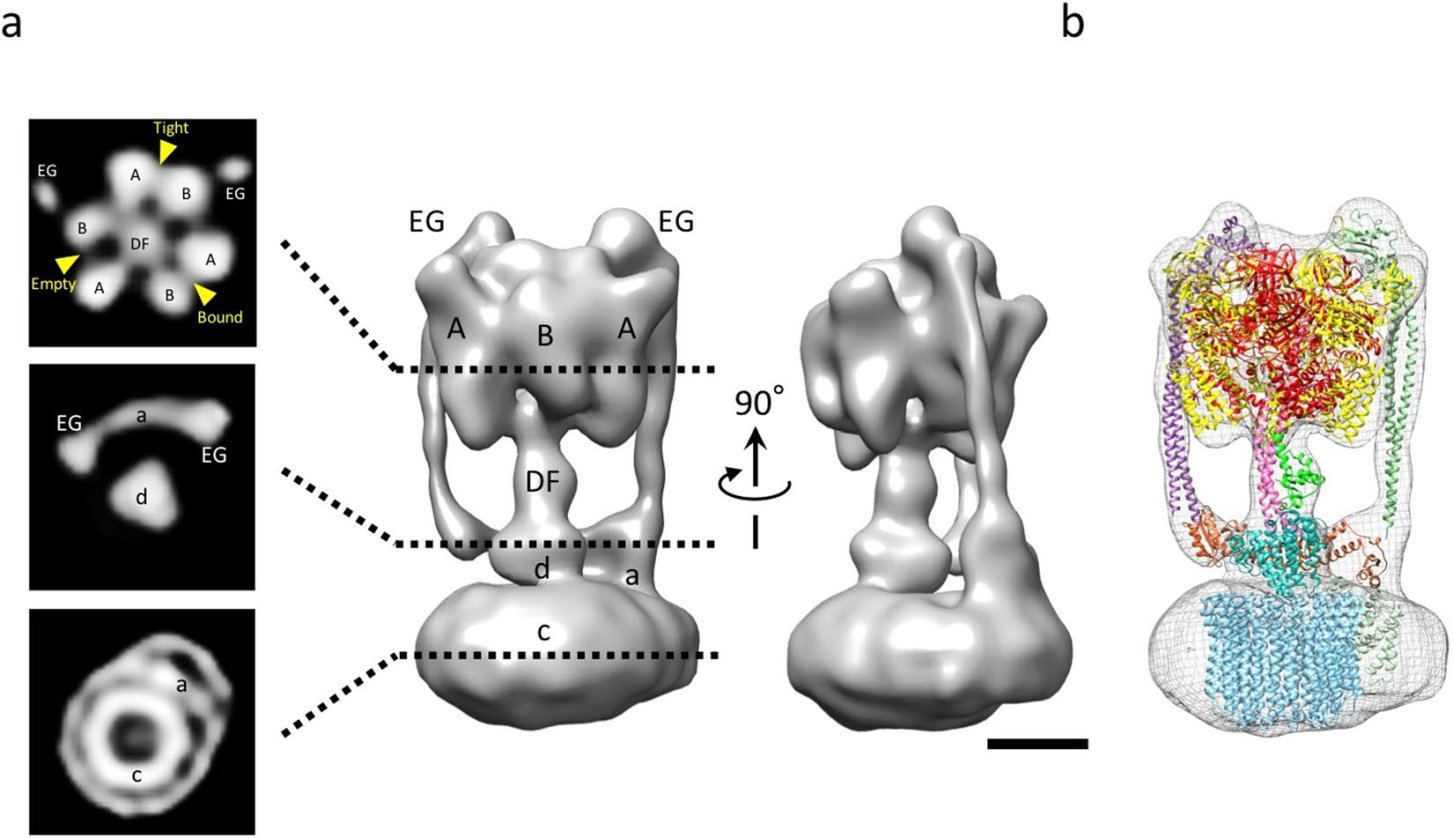
ZPP cryo-EM map of r-*Eh*V-ATPase at 17.3 Å resolution. (**a**) The cryo-EM map (right) and representative cross-sections (left): a section of cytoplasmic V_1_ domain (top panel), section of transmembrane V_o_ domain (bottom panel), and section between V_1_ and V_o_ domains (middle panel). Each subunit is labeled as A, B, DF, EG, a, d, and c. The map was separately shown with 90° different orientations. Scale bar: 5 nm. (**b**) Available atomic models were fitted into the cryo-EM map. A_3_B_3_DF (V_1_) complex from PDBID: 3VR4, EG complexes from PDBID: 3K5B (right side model; *T. thermophilus*), PDBID: 3V6I (left side model; *T. thermophilus*), c-subunit from PDBID: 2BL2, and a-subunit from PDBID: 5GAS (*T. thermophilus*). The d-subunit was created by homology modeling as a template of PDBID: 1R5Z (*T. thermophilus* d-subunit) using LOOPP server^48^.

Despite numerous attempts to find suitable detergents to stably solubilize *Eh*V-ATPase, *n*-dodecyl-β-D-maltoside (DDM) was unique to *Eh*V-ATPase. The 0.05% DDM used for solubilizing r-*Eh*V-ATPase, however, has significantly lowered the contrast of this enzyme in conventional cryo-EM (Supplementary Fig. 1), that it is very difficult to identify the individual protein particles in the images even with large defocus. We thus applied a ZPP to improve the image contrast. As shown in Fig. 2, the ZPP images clearly exhibit the particles, which will make the following single-particle analysis easier. The ZPP images were taken at in-focus condition. Therefore, the post CTF correction for each image was not applied. The particles in the uncorrected images showed the typical dumbbell-shaped structure of V-ATPase in the two-dimensional (2D) class-averages (Fig. 3a). During the reference-free 2D classification step, the featureless 2D class averages occupied by 46% of the total were removed. In the three-dimensional (3D) classification step, we initially reconstructed the 12 class 3D models (Supplementary Fig. 2), but the best resolution was achieved using all particles from 12 classes. After 3D refinement, a cryo-EM map of r-*Eh*V-ATPase was finally reconstructed with 64,158 particles at 17.3 Å resolution (Fig. 3b). The strong fringe artifacts around the 2D particle images come from the ZPP center hole were finally compensated by 3D reconstruction. The angular distribution of individual particle images showed the unbiased image sampling (Fig. 3c). An isosurface model of the cryo-EM map showed the features of the full-complex of r-*Eh*V-ATPase (Fig. 4a). The cross-sectioned images in the map revealed intercepted subunits. The cryo-EM map allowed fitting of previously reported atomic models using “Fit in Map” operation in UCSF Chimera^22^ (Fig. 4b, Supplementary Movie 1). The major structure classified represented one of three catalytic conformations of r-*Eh*V-ATPase at limited resolution, suggesting that one dominant catalytic conformation exists among three possible conformations of *Eh*V-ATPase, as also observed in *Tt*V/A-ATPase and *Sc*V-ATPase^10,11^.

### Homology modeling of the membrane-associated C-terminal half of *Eh* a-subunit

**Figure 5:**
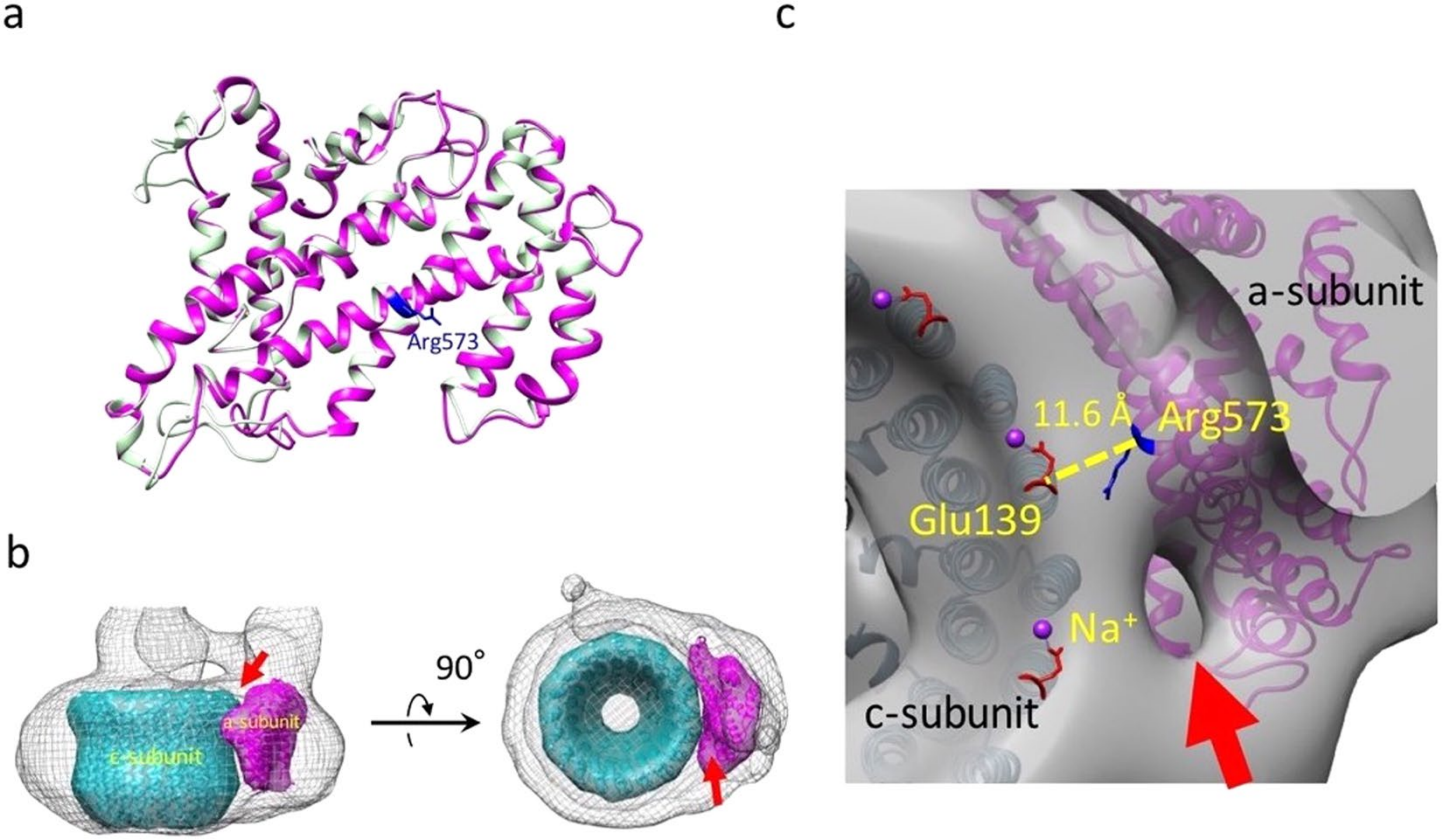
Interaction between the a- and c-subunits. (**a**) Homology model of membrane-associated C-terminal half of the *Eh* a-subunit (magenta). The original model of the *Tt* a-subunit (PDBID: 5GAS, light green) was overlaid with the homology model. The conserved arginine is labeled in blue. (**b**) The calculated maps of the a- and c-subunits were fitted into the cryo-EM map of the transmembrane V_o_ region. (**c**) Topological model between Na^+^-bound glutamate in the c-subunit (Glu139, red, PDBID: 2BL2) and conserved arginine (Arg573, blue) in the a-subunit. The closest distance between the a-subunit and c-ring, which is located around Arg573 in a-subunit and Glu139 in c-ring, is indicated. The positions of bound Na^+^ are labeled with purple spheres. A possible entrance for Na^+^ is indicated by a red arrow.

To evaluate the V_o_ domain, a model of the transmembrane C-terminal half of the *Eh* a-subunit was generated by homology modeling using SWISS-MODEL (Fig. 5a). This model was created based on the C-terminal structure of the *Tt* a-subunit (PDBID: 5GAS), which showed the highest sequence similarity to the *Eh* a-subunit (Supplementary Fig. 3). The created homology model and its calculated density were fitted into the V_o_ domain of the cryo-EM map (Fig. 5b). The closest distance between the a-subunit and c-ring was estimated as 11.6 Å, which is located around the conserved residues of Arg573 in the a-subunit and Glu139 in the c-subunit, in the fitted models (Fig. 5c). The results were not significantly different from those of other rotary ATPases; particularly, the distance was close between V-ATPases (upper three ATPases in Supplementary Table 1). We identified a small cavity on the cytoplasmic surface of the V_o_ domain in the cryo-EM map (red arrows in Fig. 5b,c). The cavity positioned between the a- and c-subunits was suggested to be the entrance for Na^+^. However, we did not find a cavity for ion release on the extracellular surface at the limited resolution.

### PA-tagged r-*Eh*V-ATPase

Our ZPP cryo-EM map showed one dominant conformation of the rotary *Eh*V-ATPase. We further adopted a peptide epitope (PA) tag system^19,20^ to initially stabilize the conformation of this enzyme, where Fab bound to the inserted PA tag in the rotor d-subunit stops the rotation of the central rotor in the rotary enzyme and the conformation is intentionally fixed in one of three catalytic states. Fab applied to the PA-tagged r-*Eh*V-ATPase was clearly identified in the purified fraction (Supplementary Fig. 4a). The activity of the ATPase was also inhibited upon Fab binding (Supplementary Fig. 4b).

**Figure 6:**
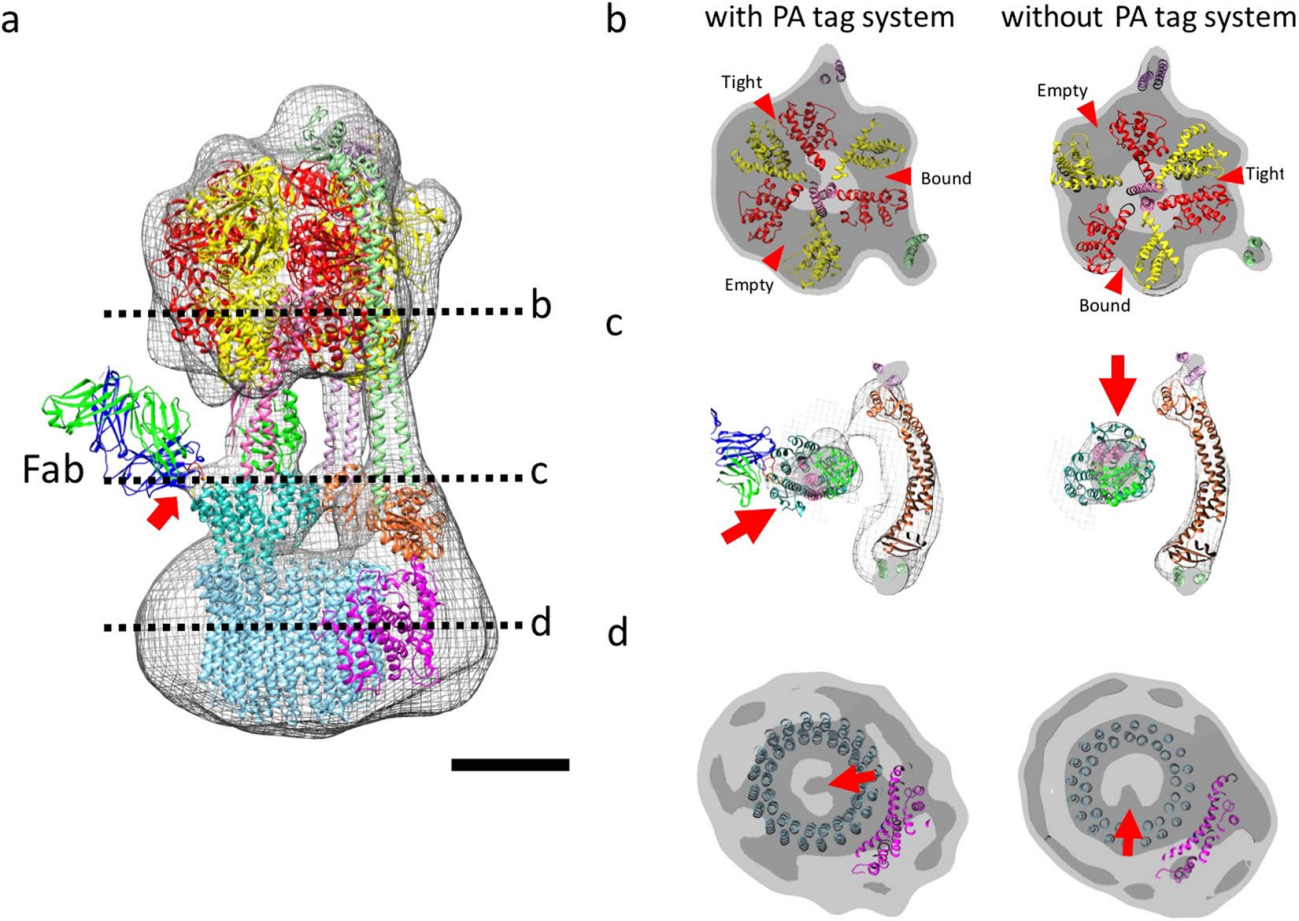
r-*Eh*V-ATPase-Fab shows a different catalytic state. (**a**) Cryo-EM map of r-*Eh*V-ATPase-Fab fitted by the atomic model of bound Fab fragment (PDBID: 4YO0). Scale bar: 5 nm. (**b**–**d**) Sections of V_1_, joint, and V_o_ regions in cryo-EM maps with and without PA tag system, whose positions are indicated in (**a**), respectively. The r-*Eh*V-ATPase-Fab shows a different state from that without the PA tag system. Red arrowheads show ATP-binding pockets. Red arrows show different orientations of the subunits with and without PA tag system.

Particles of the Fab-bound r-*Eh*V-ATPase inserted PA tag (r-*Eh*V-ATPase-Fab) were examined by single-particle ZPP cryo-EM with the same procedure as r-*Eh*V-ATPase. The particles automatically picked up from the ZPP images. In the step of the 2D class averaging, the featureless 2D class averages occupied by 69% of the total were removed (Supplementary Fig. 5a). In 3D classification step, we initially reconstructed 3 class 3D models. The best resolution was achieved when two of three classes were combined (Supplementary Fig. 6), revealing a 19.9 Å resolution structure using 24,375 particles (Supplementary Fig. 5b,c, Supplementary Movie 2). The angular distribution of individual particle images showed the unbiased image sampling (Supplementary Fig. 5d). In this map, the additional density indicating the Fab bound to the PA tag was successfully identified on the d-subunit (arrows in Fig. 6a and Supplementary Fig. 5c). However, the entire density of the Fab did not appear in the averaged map, as the opposite end of Fab bound to the PA tag was flexible.

Available models fitted into the map showed that the A_3_B_3_ complex of the V_1_ domain was fixed with another one of three catalytic states (arrowheads in Fig. 6b). The cross-sections at the junction between the V_1_ and V_o_ domains showed different rotational orientations of the DF complex and d-subunit with and without the PA tag system (arrow in Fig. 6c), while the resolutions differed between the two maps. The DF complex was rotated at 120° anticlockwise compared to that without the PA tag system viewing from the cytoplasmic V_1_ domain side, which was coupled to that of the catalytic rotation of the A_3_B_3_ subunits in the V_1_ domain (Fig. 6b). Furthermore, in the cross-sections of the V_o_ domain, a density inside the c-ring showed that the rotation of the density at 120° anticlockwise was also coupled to those of the A_3_B_3_ subunits and d subunit (arrows in Fig. 6d), although we could not determine whether the density originated from the d subunit or c-ring at the limited resolution. Interestingly, these catalytic conformations of r-*Eh*V-ATPase and r-*Eh*V-ATPase-Fab coincided with those of the minor state 2 and the major state 1 of *Tt*V/A-ATPase^10^, conversely.

### Off-axis rotor in *Eh*V-ATPase

**Figure 7:**
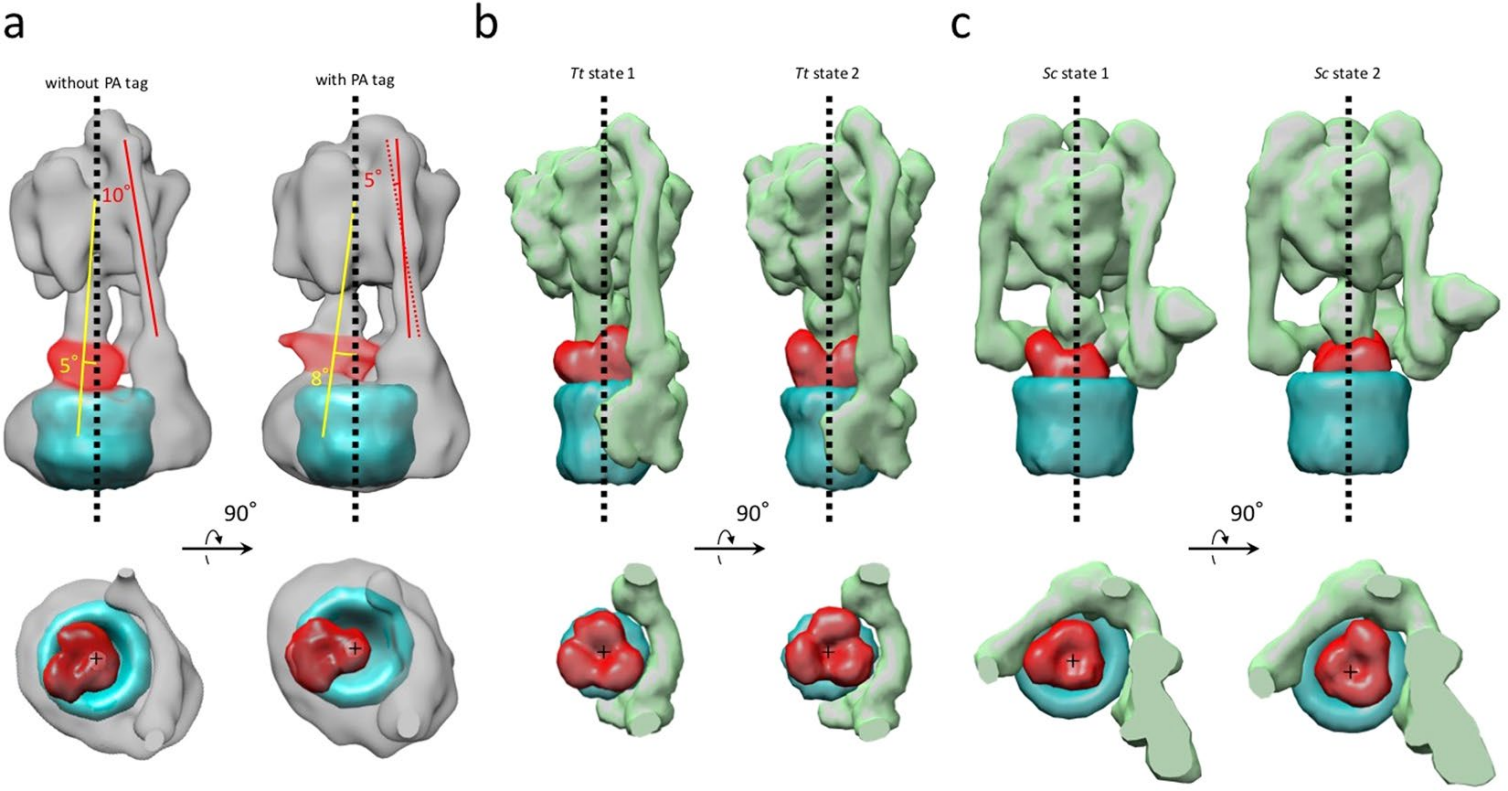
Off-axis rotor in *Eh*V-ATPase. (**a**) In *Eh*V-ATPase, the central rotors and peripheral stalks were tilted from the central axis of the c-ring (red and yellow lines). The geometric centers of the d-subunits were also shifted from the central axis of the c-ring (lower panel). (**b**) In *Tt*V/A-ATPase, the central rotors and peripheral stalks were nearly parallel to the central axis of the c-ring. The geometric centers of the d-subunits nearly coincided with the center of the c-ring (lower panel). (**c**) In *Sc*V-ATPase, the central rotors were nearly parallel to the central axis of the c-ring, though the symmetrical three peripheral stalks are tilted from the central axis of the c-ring. The geometric centers of the d-subunits nearly coincided with the center of the c-ring (lower panel).

The most prominent feature observed in *Eh*V-ATPase was the off-axis rotor, where the cytoplasmic V_1_ motor and transmembrane V_o_ pump were connected to the off-axis rotor subunits. The central rotor and peripheral stalks were tilted in *Eh*V-ATPase against the central axis of the c-ring, which were maximally 8° and 10° in our maps, respectively (Fig. 7a). The d-subunit was thus connected to an inner surface of the c-ring (lower panel in Fig. 7a). Compared to the structure with *Tt*V/A-ATPase that includes the same number of components but a smaller c-ring, the central rotor and peripheral stalks in *Tt*V/A-ATPase are nearly parallel to the central axis of the c-ring (Fig. 7b), though several peripheral stalk structures have been reported to maintain the radial wobbling of the catalytic domain during ATP hydrolysis^10,23^. Eventually, the central rotor is connected to the center of the c-ring. Further, we compared the structure with *Sc*V-ATPase, which functions as a H^+^ pump and includes the same size of c-ring with *Eh*V-ATPase but has a different number of components. In *Sc*V-ATPase, the central rotor projects to the center of the c-ring (Fig. 7c), while the symmetrical three peripheral stalks are tilted towards the central axis of the c-ring. Consequently, a combination of the large size of the c-ring and asymmetric peripheral stalks in *Eh*V-ATPase is likely to cause off-axis coupling between the V_1_ and V_o_ domains.

### Interaction between a-subunit and c-ring

**Figure 8:**
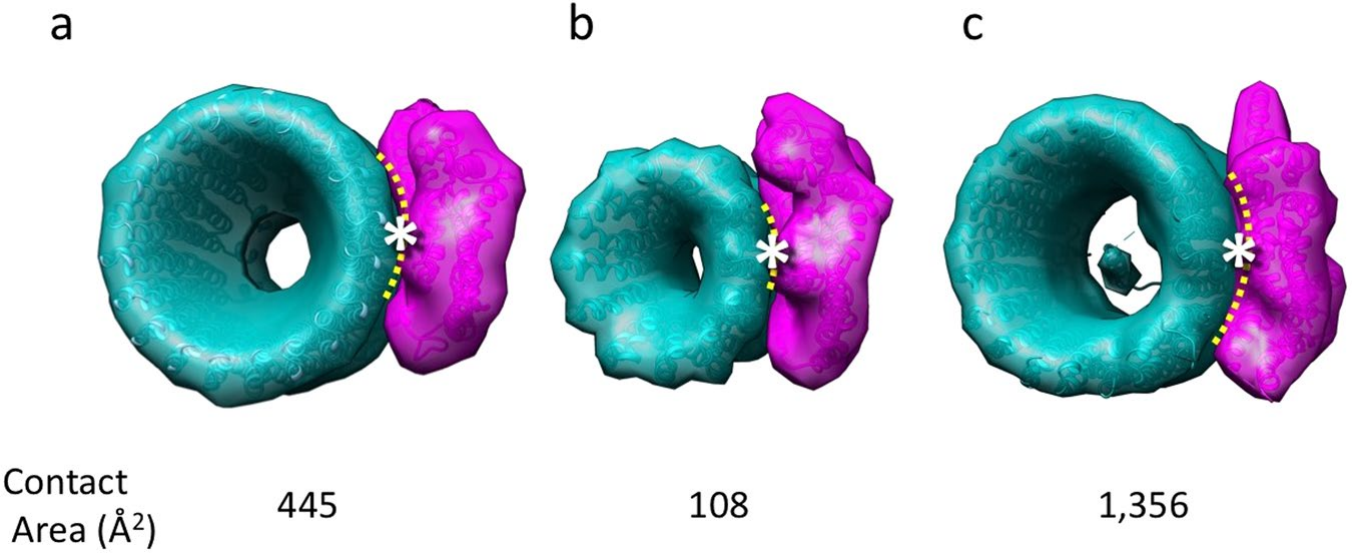
Interactions between a- and c-subunits. *Eh*V-ATPase (**a**), *Tt*V/A-ATPase (**b**), *Sc*V-ATPase (**c**). The interface sizes (indicated with yellow dotted lines) were estimated from the solvent-accessible surface areas using PyMOL software^49^. Conserved Arg573 and Glu139 are located between the a- and c-subunits (asterisks).

The size of the interface between the a-subunit and c-ring in *Eh*V-ATPase was estimated using the models, by subtracting the solvent-accessible surface area from the total surfaces of two subunits and dividing it by two. This value was then compared to those of known structures for *Tt*V/A-ATPase and *Sc*V-ATPase (Fig. 8). The interface size of 445 Å^2^ in *Eh*V-ATPase, which works for ion pump, was four-fold larger than that of *Tt*V/A-ATPase, which mainly works for ATP synthesis, though it was three-fold smaller than that of *Sc*V-ATPase (Fig. 8). The larger size of the interface between the a-subunit and c-ring may be more advantageous for ion pumping than ATP synthesis in these rotary ATPases, where it can avoid the uncoupling between ATP-driven c-ring rotation and ion transport in the case of ion pump. In contrast, the smaller size of the interface can avoid failure in the rotation of the rotor against ion transport in the case of ATP synthesis.

## Discussion

Thin-film phase plates are used with cryo-EM to visualize small and invisible protein molecules which cannot be detected by conventional cryo-EM^24–26^. Here, we applied ZPP to image the DDM-solubilized and ice-embedded *Eh*V-ATPase particles because it was difficult to stably exchange the detergent to others and to obtain sufficient contrast in DDM by conventional cryo-EM. Currently, two types of phase plate, Zernike and Volta, are adopted for cryo-EM^26^. ZPP physically exhibits relatively better contrast transfer at low spatial frequency than the Volta phase plate (VPP)^27^, although it shows strong fringe artifacts around objects come from the center hole. ZPP cryo-EM successfully enhanced the image contrast of DDM-solubilized and ice-embedded *Eh*V-ATPase particles, making it possible to process the particle images. However, the in-focus condition applied in ZPP cryo-EM hinders the substantial CTF estimation where the first zero-cross of the CTF directly determines the achievable resolution, in which an error of in-focus setting in each image severely affect the resolution^24^. In our data collected at near in-focus, an error between 500 and 5,000 Å would set the first zero crossing of CTF between 5 and 15 Å in reciprocal space^28^. It was considered to be one of the factors limiting the final resolution of the 3D reconstruction. Recently, the structure of a detergent-solubilized G protein-coupled receptor was also reported by cryo-EM using VPP^29–31^. VPP enabled collection of many images automatically, although the phase was changed during data collection^32^. Furthermore, the VPP images with a small amount of defocus enabled CTF measurement and correction, removing the resolution limit inherent to the in-focus method and allowing 3D reconstructions at near atomic resolution^33^. Our ZPP results here together with recent VPP studies^29–31^ indicate that the phase plate will be a powerful tool for single-particle cryo-EM of detergent-solubilized membrane proteins.

V-ATPases have three catalytic conformations, states 1–3, because the triplet of the A and B subunits in the V_1_ domain alternatively possess three chemical interactions with ATP known as loose, tight, and empty according to the ATP hydrolysis steps^17^. However, recent cryo-EM analyses implemented 3D classification for whole V-ATPase structures, which revealed that the ratio of the three catalytic conformations is not even. In *Sc*V-ATPase, the fractions of the three catalytic state are 47%, 36%, and 17% in states 1–3, respectively^11^. In contrast, *Tt*V/A-ATPase showed more biased fractions of 66%, 16%, and 7% in states 1–3, respectively, in addition to unclassified structures accounting for 11%^10^. Our ZPP cryo-EM map of *Eh*V-ATPase showed one major structure after 3D classification (Supplementary Fig. 2). Interestingly, this structure coincided with the minor state 2 in *Tt*V/A-ATPase based on the conformation in the V_1_ domain (Figs 4a and 6b). Furthermore, another conformation we found using the PA tag system which agreed with that of major state 1 in *Tt*V/A-ATPase. Although we have not determined the fractions of three catalytic states in *Eh*V-ATPase, these results suggest that rotary ATPases from different sources have different stable orientations of the rotor subunits, which may result in unique functions adaptive to different environments.

In this study, we determined the first whole structure of *Eh*V-ATPase which primarily pumps Na^+^. To access the mechanism of this Na^+^-specific pump, we built a homology model of the *Eh* a-subunit which was fitted into our cryo-EM map using “Fit in map” function in UCSF Chimera^22^ and evaluated the closest distance between the a-subunit and c-ring around the conserved residues of Arg573 in the a-subunit and Glu139 in the c-subunit (Fig. 5). Compared to the distance in the known structures of the six H^+^ type rotary ATPases (Supplementary Table 1), the distance of 11.6 Å in *Eh*V-ATPase is within the standard deviation of H^+^ type rotary ATPases (avg. 10.68 ± 1.27 Å). The distances were not correlated with the sizes of the c-ring. Ion selectivity of H^+^ and Na^+^ in rotary ATPases has been suggested to result from the several different amino acid residues at the ion binding site^34^. Thus, specificity is likely determined by the local environment and not the overall structure of the multi-complex.

In the transmembrane V_o_ domain, our cryo-EM map revealed a cavity on the molecular surface between the a-subunit and c-ring (Fig. 5c). This cavity only opened to the cytoplasmic surface of the V_o_ domain at the current resolution. The homology model of the a-subunit indicated that a negatively charged surface formed near the cavity, and thus we named this side as the “Entrance” of Na^+^ (Supplementary Fig. 3d). Furthermore, another negatively charged surface formed on the opposite side of the “Entrance” across the positively charged area, and thus we named this side as the “Exit” of Na^+^ (Supplementary Fig. 3d), although we did not observe the cavity in our map on the extracellular side. The positively charged area in the middle of the transmembrane a-subunit contains a conserved Arg573 residue, which plays a crucial role in Na^+^ translocation^35^. The area between the “Entrance” and “Exit” is likely to form a barrier against Na^+^ to transport ions through the c-ring rotation^36^. This characteristic surface charge distribution has also been observed in other H^+^ type rotary ATPases reported previously^9,10,37,38^. Our results suggest that the charged residues form the two half-channels in *Eh*V-ATPase as proposed previously^15^.

The most notable feature in *Eh*V-ATPase we found was the off-axis rotor, which projected from the cytoplasmic V_1_ domain into the transmembrane c-ring (Fig. 7). Compared to *Tt*V/A-ATPase, the off-axis rotor is thought to be caused by the large size of the c-ring where two peripheral stalks connecting the V_1_ domain and a-subunit, are also tilted. However, *Sc*V-ATPase showed an on-axis rotor even though the size of the c-ring was similar to that of *Eh*V-ATPase (Fig. 7c). In *Sc*V-ATPase, c″ helices located inside the c-ring may provide a scaffold for the rotor in the center of the c-ring^13^. The symmetric three peripheral stalks also function to maintain the on-axis rotor in *Sc*V-ATPase. In contrast, the empty c-ring and asymmetric two peripheral stalks in *Eh*V-ATPase may produce an off-axis rotor. Higher-resolution structures of the three catalytic conformations are needed to determine the function of the off-axis rotor.

Sequence comparison of the amino acids among V-ATPases suggested that the *Eh* a-subunit showed the highest sequence similarity to that of *Tt*V/A-ATPase, although *Tt*V/A-ATPase normally functions as ATP synthase in the bacterial membrane. In contrast, the sequence of *Sc*V-ATPase which ordinarily functions as an ion pump showed lower similarity to that of *Eh*V-ATPase, although several critical residues are preserved (Supplementary Figs 3 and 7). This suggests that the *Eh* a-subunit has a similar molecular architecture as *Tt*V/A-ATPase despite their opposite physiological functions. Comparing the structures of the V_o_ domain in *Eh*V-ATPase with other V-ATPases revealed that the combinations of the a-subunit and c-ring vary in size, as shown in Fig. 8. The interface area between the a-subunit and c-ring in *Eh*V-ATPase was four-fold larger than that in *Tt*V/A-ATPase and three-fold smaller than that in *Sc*V-ATPase. The sizes of the a-subunit and c-ring and their interface area further vary in rotary ATPases^9,29^. However, all rotary ATPases accommodate the conserved amino acids glutamate and arginine in this area with similar local distances (Supplementary Table 1). These observations suggest that rotary ATPases have adapted their robust structures to various environments during revolution.

In this study, we first determined the overall structure of *Eh*V-ATPase by single-particle ZPP cryo-EM. The major cryo-EM map classified revealed one dominant catalytic conformation of this rotary enzyme. The PA tag system introduced into the central rotor successfully identified one minor catalytic conformation. Interestingly, these structures conversely coincided with those of the minor state 2 and the major state 1 of *Tt*V/A-ATPase, respectively. As a prominent feature, *Eh*V-ATPase showed an off-axis rotor connecting the cytoplasmic motor domain and transmembrane pump domain. These unique characteristics of *Eh*V-ATPase provide new insights into rotary ATPases. Currently, the low resolution of these maps limits the understanding of the enzyme at the molecular level. Pursuit of near atomic resolution in further study will provide insight into the mechanism of rotary ATPases.

## Methods

### Expression and purification of recombinant *Eh*V-ATPase

r-*Eh*V-ATPase was expressed in *E. coli* transformed with the expression plasmid pTR19-*Eh*V-ATPase^21^. r-*Eh*V-ATPase was applied to a nickel-nitrilotriacetic acid column (Ni^2+^-NTA Superflow; Qiagen, Hilden, Germany), where the His3 tag inserted into the C-terminal of the c-subunit was used to bind r-*Eh*V-ATPase to the column. The column was equilibrated with buffer (50 mM potassium phosphate, 100 mM KCl, 5 mM MgCl_2_, 20 mM imidazole, 10% glycerol, 0.05% *n*-dodecyl-β-D-maltoside (DDM), adjusted to pH 7.5). Next, r-*Eh*V-ATPase was eluted with buffer (50 mM potassium phosphate, 100 mM KCl, 5 mM MgCl_2_, 300 mM imidazole, 10% glycerol, 0.05% DDM, adjusted to pH 7.5). The extracted r-*Eh*V-ATPases were concentrated with an Amicon Ultra 100 K unit (Merck Millipore, Billerica, MA, USA) and applied to a gel-filtration column (Superdex 200; GE Healthcare, Little Chalfont, UK) equilibrated with buffer (50 mM Tris-HCl, 5 mM MgCl_2_, 10% glycerol, 0.05% DDM, adjusted to pH 7.5).

### Cloning of r-*Eh*V-ATPase-PAtag

The plasmid pTR19-*Eh*V-ATPase^21^ was used as a template for the new construct containing the peptide epitope tag sequence (pTR19-*Eh*V-ATPase-PAtag)^19,20^. After removing the AviTag from the A subunit and His3 tag from the c-subunit in the recombinant sequence, DNA encoding a TEV protease recognition site (EHLYFQG) followed by a His6 tag was inserted into the sequence of the C-terminal of a-subunit by PCR-based mutagenesis. The DNA sequence encoding the dodecapeptide epitope tag (GVAMPGAEDDVV) was inserted into the sequence between Leu202 and Gln203 in the d-subunit by extension PCR.

### Sample preparation of r-*Eh*V-ATPase-Fab particles

r-*Eh*V-ATPase-PAtag was expressed in *E. coli* C43(DE3) transformed with pTR19-*Eh*V-ATPase-PAtag. Harvested cells were disrupted by sonication and centrifuged at 20,000 × g for 20 min at 4 °C to remove cells debris. The supernatant was ultracentrifuged at >100,000 × g for 1 h at 4 °C to collect the membrane fraction. The r-*Eh*V-ATPase-PAtag was solubilized from the membrane fraction by incubation with 2% DDM for 30 min on ice. The insoluble fraction was removed by centrifugation at >100,000 × g for 1 h at 4 °C. The solubilized membrane fraction was then applied to a Ni^2+^-NTA column (Ni^2+^-NTA Superflow, Qiagen) and eluted with buffer (50 mM Tris, 100 mM NaCl, 5 mM MgCl_2_, 300 mM imidazole, 10% glycerol, 0.05% DDM, adjusted to pH 7.5). The eluted fraction was treated with TEV protease at 23 °C for 8 h and applied to a Ni^2+^-NTA column; the digested protein was collected in flow-through fraction. The r-*Eh*V-ATPase-PAtag was then applied to a gel-filtration column (Superdex 200; GE Healthcare) equilibrated with buffer (50 mM Tris-HCl, 100 mM NaCl, 5 mM MgCl_2_, 10% glycerol, 0.05% DDM, adjusted to pH 7.5). The purified r-*Eh*V-ATPase-PAtag was concentrated and incubated with a 4-fold molar excess of the NZ-1 Fab fragment for 30 min at 4 °C. The mixture was then applied to a gel filtration column (Superdex 200, GE Healthcare) equilibrated with buffer (50 mM Tris, 100 mM NaCl, 5 mM MgCl_2_, 10% glycerol, 0.05% DDM, adjusted to pH 7.5). The fractions of r-*Eh*V-ATPase-Fab particles were concentrated to 1.2 mg/mL after confirmation by SDS-PAGE.

### Zernike phase plate cryo-electron microscopy

Purified r-*Eh*V-ATPase and r-*Eh*V-ATPase-Fab were applied to a Quantifoil R1.2/1.3 Mo grid (Quantifoil Micro Tools GmbH, Großlöbichau, Germany) that had previously been glow-discharged, and quickly frozen in a liquid ethane using Vitrobot Mark IV (FEI Company, Hillsboro, OR, USA). Frozen grids were imaged with a JEM-2200FS operating at 200 kV accelerating voltage equipped with an omega-type energy filter and field emission electron source (JEOL Ltd., Tokyo, Japan). The images were recorded on a DE20 direct detector (Direct Electron LP, San Diego, CA, USA). Zernike phase plates (ZPP) prepared in our laboratory were inserted into the back focal plane of the objective lens^39^. The electron dose was below 20 e^−^/Å^2^ for each image. Images consisting of movie frames were collected with a numerical pixel size corresponding to 1.992 Å on the specimen. Movie frames were motion-corrected using a manufacture-provided script, DE_process_frames.py, and summed.

### Single-particle analysis

A total of 119,481 r-*Eh*V-ATPase particles were picked from 665 motion-corrected images using RELION-2 software^40,41^. The extracted particles were classified by reference-free alignment. A total of 64,158 particles were selected from well-aligned 2D classes and used for 3D map refinement. As the initial model, two spheres located at the same distance between the V_1_ and V_o_ domains were geometrically generated and used. The final map resolution was estimated using the Gold-standard Fourier shell correlation (GS-FSC = 0.143) criterion. Next, 114,509 r-*Eh*V-ATPase-Fab particles were picked from 1,302 images and 2D-classified using the same procedures described above. We selected 35,413 particles in well-aligned 2D classes and subjected these particles to 3D classification. The finally selected 24,375 particles were used for 3D map refinement. The cryo-EM maps were visualized and annotated by UCSF Chimera^22^.

### Homology model building of the a-subunit

Sequence similarity of the *Eh* a-subunit was searched by protein-protein BLAST in the PDB^42^. As a result, the C-terminal half of *Tt* a-subunit showed the best similarity score with the *Eh* a-subunit (amino acids of 318–655, Score 103 bits, E-value = 3e^−27^). Transmembrane helices of *Eh* and *Tt* a-subunits were predicted by TransMembrane hidden Markov model (TMHMM)^43^. A homology model of the C-terminal half of the *Eh* a-subunit was built using SWISS-MODEL^44–47^.

## Electronic supplementary material

Supplementary information

Supplementary Movie 1

Supplementary Movie 2

## Data Availability

Cryo-EM maps of r-*Eh*V-ATPase and r-*Eh*V-ATPase-Fab have been deposited into the Electron Microscopy Data Bank under accession numbers of EMD-9661 and EMD-9662, respectively.
