# Supplementary material for: Off-axis rotor in Enterococcus hirae V-ATPase visualized by Zernike phase plate single-particle cryo-electron microscopy
Source: Sci Rep. 2018 Oct 23;8:15632. doi: 10.1038/s41598-018-33977-9 (PMC6199243; doi:10.1038/s41598-018-33977-9)
Supplement: Supplementary file 1 — Supplementary information [file 41598_2018_33977_MOESM1_ESM.pdf]

Supplementary information

Off-axis rotor in *Enterococcus hirae* V-ATPase visualized by Zernike phase plate single-particle cryo-electron microscopy

Jun Tsunoda[1,2], Chihong Song[2], Fabiana Lica Imai[3], Junichi Takagi[4], Hiroshi Ueno[5], Takeshi Murata[3,6], Ryota Iino[1,7], Kazuyoshi Murata[1,2*]

[1] The Graduate University for Advanced Studies (SOKENDAI), Kanagawa, 240-0193, Japan

[2] National Institute for Physiological Sciences, Okazaki, Aichi, 444-8585, Japan

[3] Department of Chemistry, Graduate School of Science, Chiba University, Inage, Chiba, 263-8522, Japan

[4] Institute for Protein Research, Osaka University, 3-2 Suita, Osaka, 565-0871, Japan

[5] Department of Applied Chemistry, University of Tokyo, Tokyo, 113-8656, Japan

[6] JST, PRESTO, Inage, Chiba, 263-8522, Japan

[7] Institute for Molecular Science, Okazaki, Aichi, 444-8787, Japan

[*]Correspondence to: kazum@nips.ac.jp

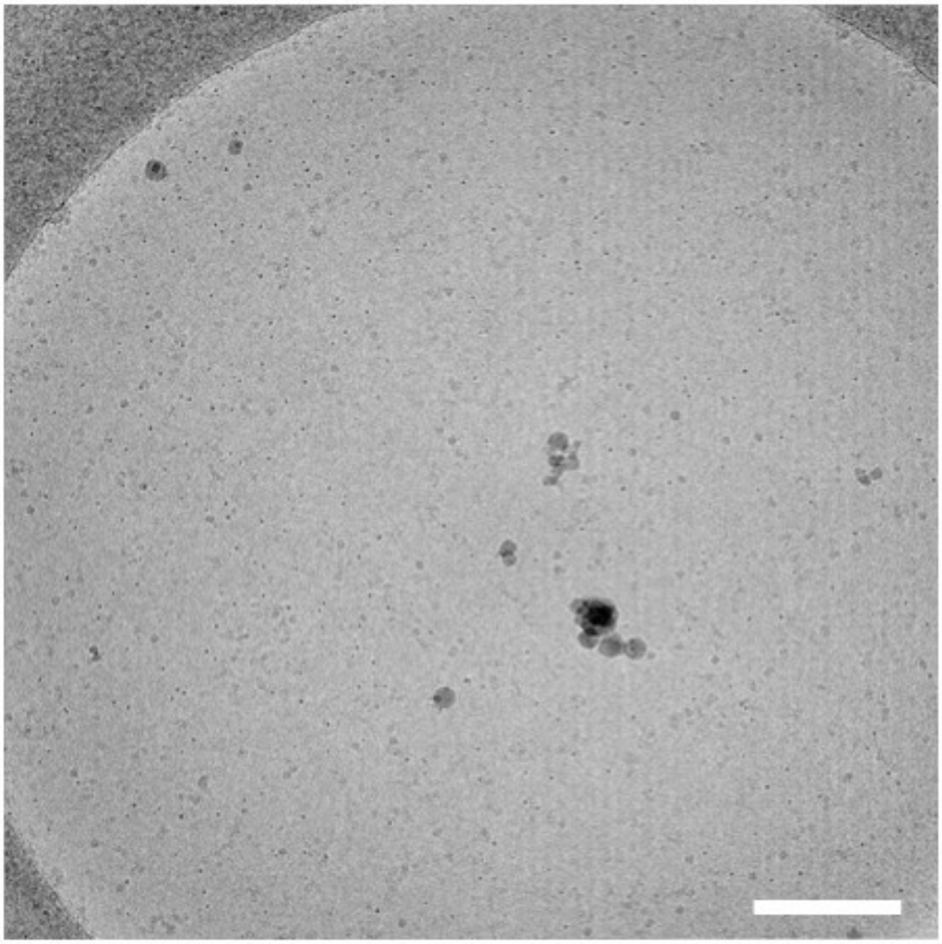

Supplementary Figure S1. r-*Eh*V-ATPase image by conventional cryo-EM. The image was acquired under the same image conditions as that in Fig. 2A. The low-contrast images made it difficult to identify the particles. Scale bar: 200 nm.

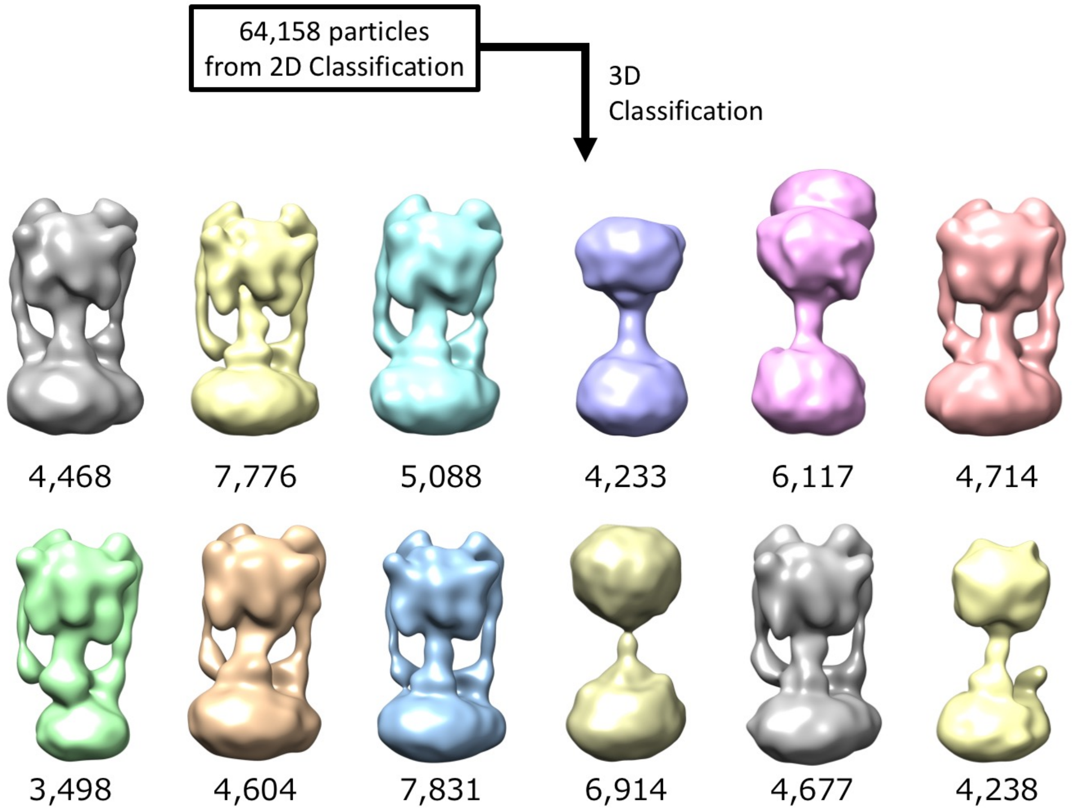

Supplementary Figure 2. 3D Classification of r-*Eh*V-ATPase. 64,158 particles selected from well-aligned 2D classes were reconstructed at 3D and classified into 12 classes. The best resolution was achieved when all classes were combined. The number of particles contained each class were indicated.

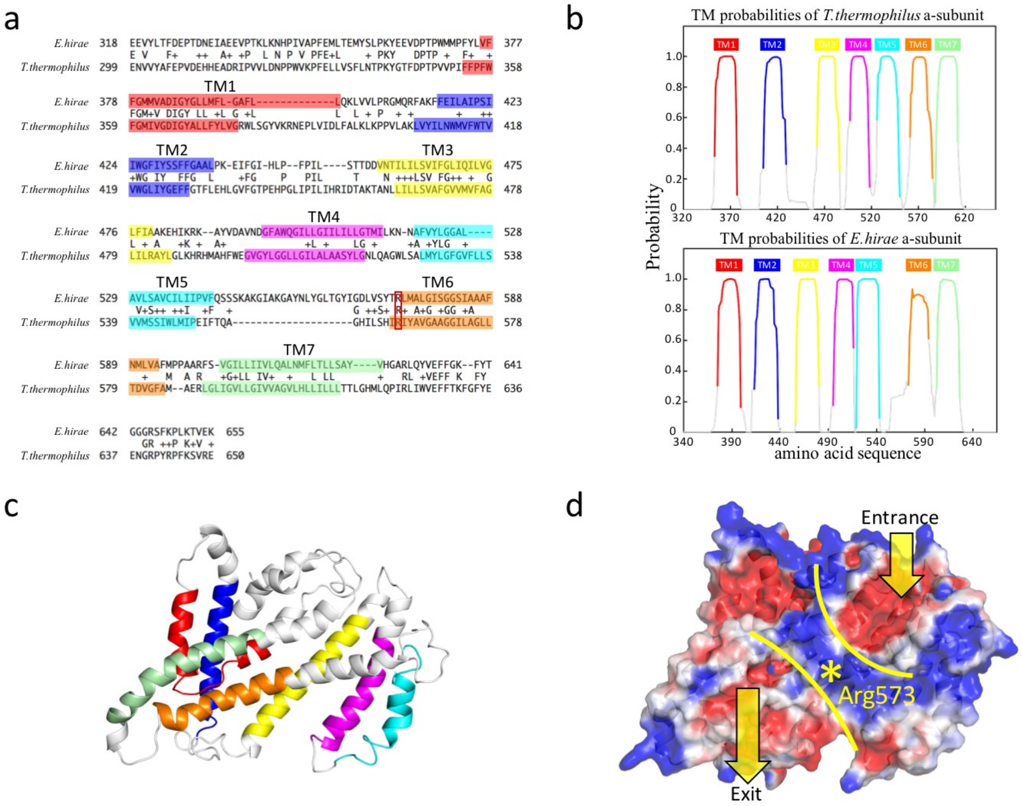

Supplementary Figure 3. Homology modeling of the membrane-associated C-terminal half of *Eh* a-subunit. a) Sequence comparison of the membrane-associated C-terminal half of the a-subunit between *E. hirae* and *T. thermophilus*. The two sequences showed 29% identity and 45% similarity by BLAST search. Predicted TM regions are highlighted in colors, and the conserved Arg is boxed in red. b) Predicted transmembrane regions in a-subunits of *E. hirae* (upper panel) and *T. thermophilus* (lower panel) by TMHMM. c) Predicted TM regions are labeled with colors on the ribbon diagram of the homology model. d) Electrostatic potential surface of the homology model calculated by Adaptive Poisson-Boltzmann Solver (APBS)[1,2]. Positively and negatively charge residues are colored in blue and red. The expected entrance and exit of Na^+ are indicated with yellow arrows. The location of Arg573 is labeled. The large positively charged area is indicated with yellow lines. C) and D) were prepared using the PyMOL Molecular Graphics System, Version 2.0 Schrödinger, LLC.

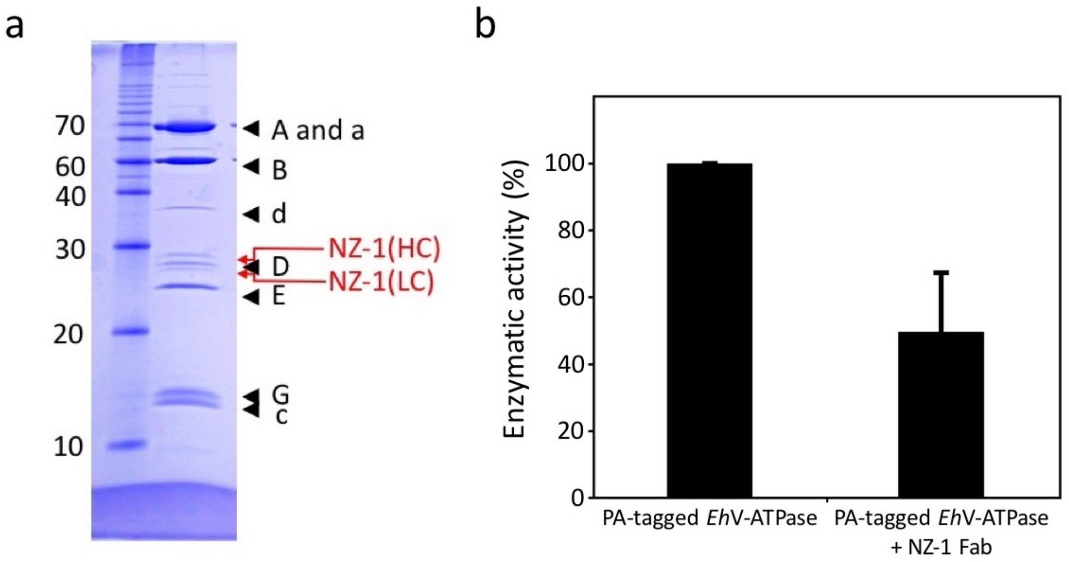

Supplementary Figure 4. Preparation of r-*Eh*V-ATPase-Fab. a) SDS-PAGE of PA-tagged *Eh*V-ATPase with Fab fragment of NZ-1. HC: heavy chain of NZ-1, LC: light chain of NZ-1. The full-length gel is presented. b) Inhibition curve of ATPase activity with Fab fragment of NZ-1 (n=3). The standard errors are included in the bars.

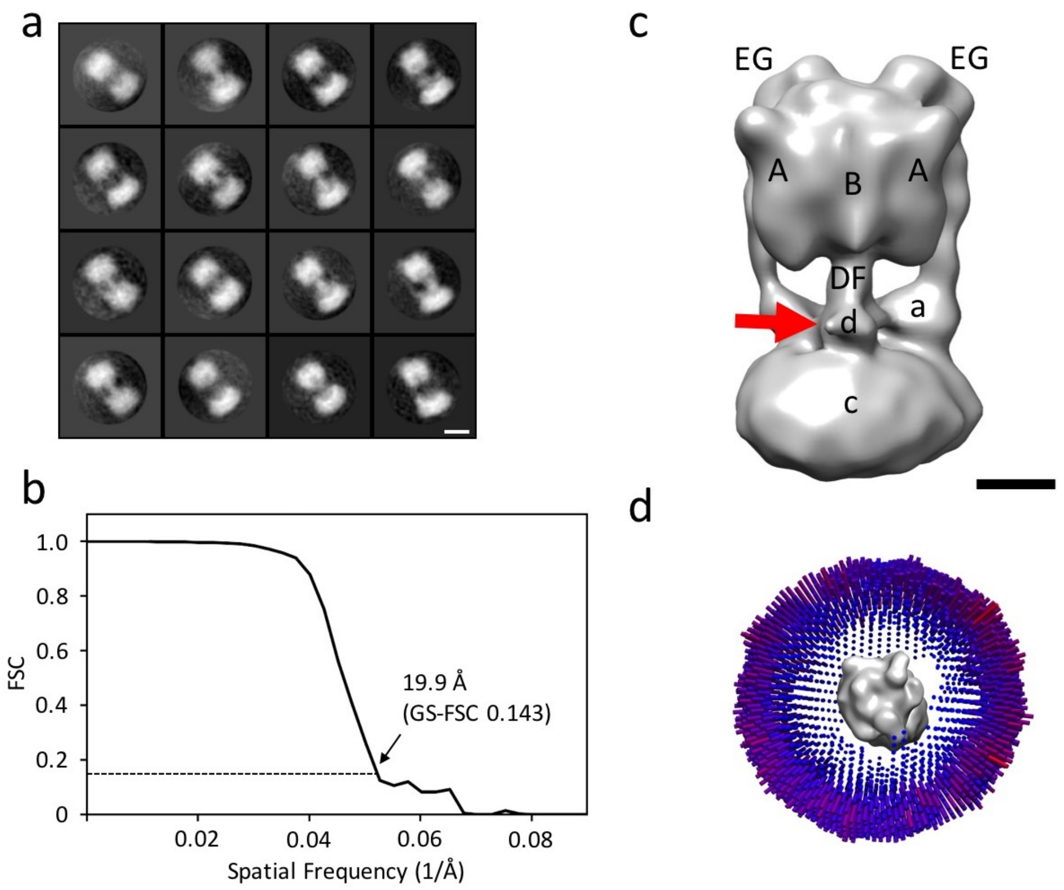

Supplementary Figure 5. Single particle analysis of r-*Eh*V-ATPase-Fab by ZPP cryo-EM. a) Representative 2D class averages of r-*Eh*V-ATPase-Fab particles. Scale bar: 10 nm. b) Gold-standard Fourier shell correlation (GS-FSC) curve for the final cryo-EM map of r-*Eh*V-ATPase-Fab. Resolution of the cryo-EM map was estimated as 19.9 Å using GS-FSC criterion. c) The cryo-EM map of r-*Eh*V-ATPase-Fab. Additional density is identified on the d-subunit (arrow). Individual subunits are labeled on the map. Scale bar: 5 nm. d) Angular distribution of individual images in the final 3D reconstruction, showing unbiased image sampling was achieved.

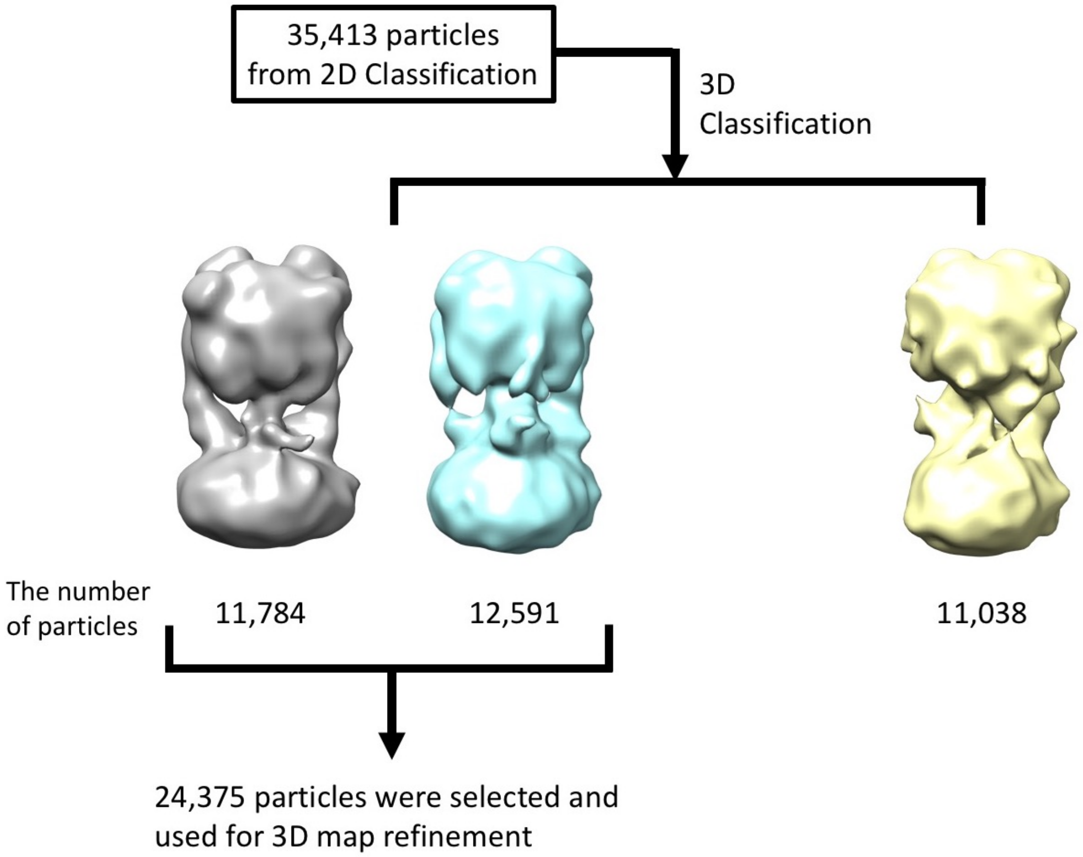

Supplementary Figure 6. 3D Classification of r-*Eh*V-ATPase-Fab. 24,375 particles selected from well-aligned 2D classes were reconstructed at 3D and classified into 3 classes. The best resolution was achieved when two of three classes were combined. The number of particles contained each class were indicated.

a

```
E. hirae       232  QQQLVAAKQSLQEI----KDQQKKLSSAIGACSGYIKDFEWTEEIFLARSEREAIKDRII  287
                     QQL    ++L ++     K     L S + A +  +    W +++    ++  E +
S. cerevisiae  271  SQQLAKVNKNLSDLYTVLKTTSTTLESELYAIAKELD--SWFQDVTREKAIFEILNKSNY  328

E. hirae       288  HTPYLILI-QGWVDHEEKQELIHMLQNILASEEVYLTFDEPTDNEIAEE---VPTKLKNH  343
                     T   ILI +GW+  +E    L    L  ++A      L  D P+  ++ +      PT  + +
S. cerevisiae  329  DTNRKILIAEGWIPRDELATLQARLGEMIAR----LGIDVPSIIQVLDTNHTPPTFHRTN  384

E. hirae       344  PIVAPFEMLTEMYSLPKYEEVD---PTPWMMPFYLVFFGMMVADIGYGLLMFLGAFLL--  398
                       A F+ + + Y + +Y E++    PT     PF     F +M   D+G+G LM L A  L
S. cerevisiae  385  KFTAGFQSICDCYGIAQYREINAGLPTIVTFPF---MFAIMFGDMGHGFLMTLAALSLVL  441

E. hirae       399  --QKLVVLPRG----MQRFAKFFEIL-AIPSIIWGFIYSSFFGAAL  437
                     +K+  + RG     M     ++  +L  + S+  GF+Y+  F   +
S. cerevisiae  442  NEKKINKMKRGEIFDMAFTGRYIILLMGVFSMYTGFLYNDIFSKTM  487
```

b

```
                             ▼
E. hirae       565  IGDLVSYTRLMALGISGGSIAAAFNMLVAFMPPAARFSVGILLIIVLQALNMFLTL----  620
                    +     SY RL AL ++     +++     +    +     R  VG+ + + L A+    LT
S. cerevisiae  727  VSHTASYLRLWALSLAHAQLSSVLWTMTIQIAFGFRGFVGVFMTVALFAMWFALTCAVLV  786

                                ▼
E. hirae       621  ----LSAYVHGARLQYVEFFGKFYTGGGRSFKPL  650
                         SA +H   RL +VE   KF+ G G  ++P
S. cerevisiae  787  LMEGTSAMLHSLRLHWVESMSKFFVGEGLPYEPF  820
```

Supplementary Figure 7. Sequence comparison of the a-subunit between *E.hirae* and *S. cerevisiae* by BLAST search. a) Similarity was detected in a joint between the N-terminal region and C-terminal region. The two sequences showed 24% identity and 43% similarity. b) Similarity was particularly high for the last two helices of the a-subunit. This result shows 27% identity and 44% similarity. Conserved Arg residues in the α-helices are indicated by red arrowheads.

Supplementary Table 1. The closest distance between the a-subunit and c-ring in structurally-known ATPases.

ATPases	The closest distance between the a-subunit and c-ring (Å)	Character of c-ring			PDBID
		The number of transmembrane helices	Diameter (nm)	Ion type	
E. hirae V-ATPase	11.6	40	8	Na^+	
T. thermophilus V/A-ATPase	10.1 (Arg563 - Glu63)	24	6.5	H^+	5GAS
S. cerevisiae V-ATPase	11.5 (Arg735 - Glu108 in c")	40	8	H^+	5TJ5
E. coli ATP synthase	11.6 (Arg210 - Glu61)	20	5	H^+	5T4O
P. denitrificans ATP synthase	8.6 (Arg182 - Glu60)	24	6	H^+	5DN6
P. angusta F-ATPase	10.3 (Arg179 - Glu59)	20	5	H^+	5LQZ
Bovine mitochondrial ATP synthase	12.0 (Arg158 - Glu58)	16	4	H^+	5ARA

Supplementary Movie 1: ZPP cryo-EM map of *r-Eh*V-ATPase at 17.3 Å resolution. The cryo-EM map allowed fitting of previously reported atomic models.

Supplementary Movie 2: ZPP cryo-EM map of *r-Eh*V-ATPase-Fab at 19.9 Å resolution. The cryo-EM map allowed fitting of previously reported atomic models including Fab.

REFERENCES

1. Baker, N. A., Sept, D., Joseph, S., Holst, M. J. & McCammon, J. A. Electrostatics of nanosystems: Application to microtubules and the ribosome. *Proc. Natl. Acad. Sci.* **98**, 10037–10041 (2001).
2. Dolinsky, T. J., Nielsen, J. E., McCammon, J. A. & Baker, N. A. PDB2PQR: An automated pipeline for the setup of Poisson-Boltzmann electrostatics calculations. *Nucleic Acids Res.* **32**, 665–667 (2004).
